# From human teams to hybrid intelligence teams: identifying, characterizing, and evaluating foundational quality attributes

**DOI:** 10.1007/s10458-025-09730-8

**Published:** 2026-02-18

**Authors:** Davide Dell’Anna, Pradeep K. Murukannaiah, Mireia Yurrita, Bernd Dudzik, Davide Grossi, Catholijn M. Jonker, Catharine Oertel, Pınar Yolum

**Affiliations:** 1 Utrecht University, Utrecht, The Netherlands; 2 Delft University of Technology, Delft, The Netherlands; 3 University of Groningen, Groningen, The Netherlands; 4 University of Amsterdam, Amsterdam, The Netherlands; 5 Leiden University, Leiden, The Netherlands; 6 Vrije Universiteit Amsterdam, Amsterdam, The Netherlands

**Keywords:** Hybrid Intelligence, Quality model, Human-agent teamwork, Sociotechnical systems, Team Diagnostic Survey, Competitions

## Abstract

Hybrid Intelligence (HI) is an emerging paradigm in which artificial intelligence (AI) augments human intelligence. The current literature lacks systematic models that guide the design and evaluation of HI systems. Further, discussions around HI primarily focus on technology, neglecting the holistic human-AI ensemble. In this paper, we take the initial steps toward the development of a quality model for characterizing and evaluating HI systems from a human-AI teams perspective. We first conducted a study investigating the adequacy of properties commonly associated with effective human teams to describe HI. The study features the insights of 50 HI researchers, and shows that various human team properties, including boundedness, interdependence, competency, purposefulness, initiative, normativity, and effectiveness, are important for HI systems. Based on these results, we developed a quality model for HI teams composed of seven high-level quality attributes, further refined into 16 specific ones. To evaluate the relevance and understanding of the proposed attributes, we conducted a second empirical investigation by staging competitions in which participants used the quality model to develop and analyze HI usage scenarios. Our analysis of 48 collected scenarios, which we openly release, confirms the proposed attributes’ relevance and highlights insights that emerge when designers consider the quality model in HI system design.

## Introduction

Artificial Intelligence (AI) permeates many aspects of our daily lives. Literature suggests that AI should work synergistically with humans instead of replacing them to benefit individuals and society [2, 127]. The concept of *Hybrid Intelligence* (HI) [2, 32], in particular, argues for a combination of human and artificial intelligence instead of their isolated operations. Various interpretations of HI exist in the literature, including: HI as an emergent property of human-machine interactions [16, 111, 123], HI as a form of human-in-the-loop or AI-in-the-loop decision making [94, 125], HI as a type of collective intelligence [16], and HI as a design paradigm [18, 89, 99, 144], just to mention a few.

Despite such a wide array of interpretations, to date there exists no quality model [139] that guides the systematic development and evaluation of HI systems [83]. Quality models (e.g., [68]) are conceptual representations of quality characteristics of a product, e.g., a software or a system (in our case a HI system). Quality models play a critical role in assuring product quality, i.e., the degree to which a product satisfies stated and implied stakeholder needs [7, 139]. Poor product quality and the lack of quality assurance techniques based on well-defined quality attributes, have been shown to result in highly adverse and costly outcomes across sectors [40, 82, 122, 138]. In the context of HI systems, where humans play a central role, adverse outcomes can be high-stake.

Existing work on HI adopts a technology-centric perspective [31, 61, 113, 145], delineating requirements and attributes of AI components but neglecting the system-level view that underpins crucial concepts of HI such as collaboration, synergy, interdependence, and human-AI relationship. Similarly, guidelines and models for quality AI systems (e.g., [45, 48, 53, 126]) mainly address the engineering of AI components. In contrast, we emphasize the *human-AI system* and adopt a team-oriented approach, framing HI systems as human-AI teams (or HI teams) [2, 60, 124, 143]. The teaming perspective allows us to explore human- and system-centric dimensions of HI and go beyond existing technology-oriented views.

### Aim of the paper

The aim of this paper is to articulate an empirically grounded quality model for HI. To do that we address two groups of research questions.

The *first group* of questions is geared towards identifying and characterizing two key elements of a quality model for HI teams: quality *attributes* and quality *measures* [108, 139].

*RQ1 (Adequacy)*: To what extent are the properties of human teams adequate to characterize HI teams?
*RQ2 (Effectiveness)*: Which measures of effectiveness of human teams are also important for HI teams?

We design and conduct an empirical study to answer these research questions. Through group discussions and a hands-on exercise, 50 HI researchers evaluated various properties and effectiveness measures derived from the *Team Diagnostic Survey* (TDS) [137], a well-known instrument employed in practice to diagnose the strengths and weaknesses of human teams.

Our findings show that HI experts consider several properties of effective human teams important for HI teams. Some of these properties are well-understood and directly applicable to characterize HI teams. These encompass the concepts of well-defined and interdependent teams, appropriate team size, members diversity, clear meaningful mission, member autonomy and initiative, and team norms. Further, our study indicates that seven measures of human teams effectiveness, encompassing task performance, quality of group processes, and member satisfaction, are deemed important criteria for evaluating HI teams effectiveness.

Based on the above findings, in [28], we proposed seven high-level quality attributes for a quality model for HI teams, further refined into 16 specific ones, which led us to the *second group* of research questions geared towards evaluating the *understanding* of the quality model and the *relevance* of the proposed attributes. The present paper extends our earlier work by developing empirically-informed answers to the following questions:

*RQ3 (Understanding)*: How is the quality model understood when applied to HI Teams?
*RQ4 (Relevance)*: To what extent are the proposed quality attributes relevant for HI Teams?

To answer RQ3 and RQ4, we collect data by running three competitions^1^ at two international AI conferences, namely AAMAS 2024 and HHAI 2024, and at the 13th Dutch Hybrid Intelligence consortium meeting. The competitions, focused on “foundational qualities of HI teams”, led to the generation of a data set composed of 48 HI scenarios, which we analyze to answer RQ3 and RQ4. Our evaluation provides interesting insights about the quality model, such that some quality attributes (e.g., transparency of objectives) are perceived as natural elements of teamwork, whereby their absence jeopardizes teamwork, some quality attributes (e.g., autonomy) are perceived to be significant when used for AI components of the team, rather than their human counterparts, and effectiveness attributes are perceived to characterize the output of the team, rather than the team processes.

### Paper contribution and structure

HI is an emerging field, and understandably, there is a lack of construct clarity [131]. Our work conceptualizes *quality* in HI teams by offering a series of observations and recommendations derived from a systematic study involving HI and AI researchers. Further, we show how the insights from our study can be used to create a quality model, consisting of quality attributes and measures, for HI teams. Such a model can help analyze existing HI teams by diagnosing missing attributes or performance problems, useful to improve HI teams over time. The model can also enable quality-driven design and development of HI teams from the onset. Finally, we describe the design and execution of the 1st Hybrid Intelligence Competition, and openly release a novel dataset of 48 HI scenarios for researchers and practitioners to guide the development and evaluation of HI teams.

The paper is organized as follows. Section 2 discusses related work. Section 3 provides a background on TDS and addresses RQ1 and RQ2. Section 4 describes the proposed quality model for HI teams. Section 5 addresses RQ3 and RQ4. Section 6 provides discussion and conclusions.

## Related work

The ideas underlying the concept of HI, sometimes referred to as *human-machine intelligence* or *human-machine symbiosis* [62, 75, 83] root back to the 50s [42, 88]. The term HI was first used in the late 70s in contexts of cybernetics [90] and ergonomics [20]. Nearly four decades later, the concept of HI has found a variety of interpretations and applications in various areas, including education [101], medicine [86], healthcare [36], and computer vision [145].

Akata et al. [2], for example, define hybrid intelligence as “the combination of human and machine intelligence, augmenting human intellect and capabilities instead of replacing them and achieving goals that were unreachable by either humans or machines”. Dellerman et al. [31], list, among others, the following systems as empirical evidence of Hybrid Intelligence from recent business applications: Teachable machine, Vencortex, Cobi, AlphaGo, and the Mirosoft Project Custom Decision. Molenaar et al. [101] introduce an Adaptive Learning Technology as an example of hybrid intelligence system for teaching students self-regulating learning based on their performance. Lee et al. [86] presents the Stargazer interactive dashboard that facilitates rapid investigation of potential novel drug targets as an hybrid intelligence platform. Zschech et al. [145] introduce Computer Vision-based Hybrid intelligence systems. Greeff et al. [51] discuss the FATE (FAir, Transparent and Explainable) hybrid intelligence, a system aimed at providing decision support to the humans.

Various taxonomies organize knowledge around HI and provide a framework for their design and evaluation [61, 94]. Dellerman et al. [31] characterize HI via four dimensions: task characteristics, learning paradigm, human-AI interaction, AI-human interaction. These dimensions are (AI) task-oriented, e.g., they identify four task categories: recognition, prediction, reasoning and action. Pescetelli [113] indicates that HI has various levels related to the algorithm’s role, e.g., the AI being an assistant, a peer, a facilitator, or a system-level operator. Zschech et al. [145] define design principles for computer vision-based HI, focusing on the AI capabilities. A similar focus on technology appears in recent initiatives delineating guidelines for ensuring AI systems quality (e.g., [45, 48, 126]). Kuwajima et al. [85] integrate EU Guidelines for Trustworthy AI [19] into the ISO25010 [68] standard for system and software quality models. They propose to use quality characteristics, such as controllability, explainability, collaboration effectiveness, privacy, human autonomy risk mitigation and unfair bias risk mitigation. Habibullah et al. [53] identify quality requirements for machine learning, such as transparency and explainability. These works align with our objective of ensuring trustworthiness of Human-AI systems [41, 80] by providing systematic guidelines (e.g., via quality models) for their development. However, the focus on AI components of current literature can downplay the crucial concepts for HI, such as collaboration, synergy, interdependence, and relationship between human and AI agents [2, 50, 141]. This motivates the human-agent team orientation of our work, complementing the existing literature.

There is extensive literature on teamwork (e.g., [10, 12, 106, 132]) and human-AI (including, human-robot and human-agent) teams [77, 110, 123]. Johnson et al. [73] provide a design-time approach for handling interdependence between actors of a team that can be translated into control algorithms. Bansan et al. [5] study how adaptation of agents at run-time affect the interactions between humans and agents. Zhang et al. [143] investigate how complementary expertise between humans and agents play a role in creating team trust, and Kox et al. [81] develop strategies for agents to repair trust in human-AI teams. Wang et al. [140] design agents that provide explanations for their decisions to establish trust from humans in teams. Georgara et al. [46] develop explanation algorithms to clarify why certain teams can be formed with team formation algorithms and others cannot. Gervits et al. [47] make use of shared mental models to improve performance of human-robot teams. Paynadath et al. [115] design an agent that considers team-level properties when making interventions to a human team.

These works often take inspiration from the prolific literature on human teams, which have been studied from many angles [52, 97]. Broadly, a team involves two or more *members* working *interdependently* toward a *common goal* [119]. Teams are generally characterized by a collective identity that implies that members share a common (team) goal and that team activities result in collective (as opposed to individual) success or failure. Activities in a team require *mutual accountability* from all members.

IMO is a well-known conceptual model for teamwork [66, 96]. In IMO, the I represents *team inputs* (team composition, tasks complexity, members’ differences), which are converted by *team mediators* (M) into *team outputs* (O)—team’s outcomes such as team viability, individual learning, development and satisfaction [54]. Mediators include team processes [14, 93] and emergent states [22, 66, 79]. Mark [93] distinguishes three types of activities that characterize team processes. Transition-related activities involve developing, adapting, and clarifying the team’s common purpose, strategy, tasks, and role structure. Action-related activities involve back-up behaviors, mutual performance monitoring, monitoring of goal progression, and coordination that occurs during task execution periods. Interpersonal activities involve affect management, conflict management, and motivational issues. Emergent states [66, 79] refer to affective or cognitive qualities of the team, such as trust and shared mental models.

Many human team design and assessment tools are inspired from the IMO model. In a systematic literature review, Valentine et al. [133] report 39 surveys that measure features of team’s design, teamwork and team effectiveness [21]. The Team Diagnostic Survey (TDS) [137], designed to diagnose the strengths and weaknesses of a team, is one such instrument. The extensive validation of TDS [133, 137] has shown that TDS provides a good trade-off between simplicity, abstractness and coverage of concepts concerning human teams. For this reason, in this paper, we use TDS as a starting point for characterizing HI from a human-AI teams perspective. We provide more details about TDS in Section 3.1.

The works above provide a fertile ground for research on human-AI teams and HI by considering team aspects such as trust [5, 81, 100, 140], team cohesion [134], proactivity and shared mental models [38, 47], interdependence and joint activities [73, 77], and team performance [115]. However, there is still a lack of studies that pinpoint desired properties that HI teams should exhibit, and instruments that capture these properties into quality models with which teams can be analyzed. As a step in this direction, we start from TDS as a human team model, investigate its applicability to HI teams [118], and develop a system-oriented, quality model for HI teams.

## Foundations for a quality model for HI teams

In this section, we describe the design and execution of an empirical study to answer research questions RQ1 and RQ2. Our study seeks to elicit expert knowledge by facilitating discussions among the participants to yield diverse perspectives, and promote iterative ideas refinement before the participants answer a questionnaire.

### Team Diagnostic Survey

A key objective for our study is to foster a human-centered discussion of human-AI teams as opposed to technology-oriented discussions. Thus, we took the Team Diagnostic Survey (TDS) as a starting point for the discussions on relevant properties of HI teams. In addition to its practical relevance, TDS is strongly human-oriented. This encouraged discussions on the applicability of human aspects of teamwork to HI teams. Table 1 provides an overview of TDS.

**Table 1:** Overview of the *properties* composing the Team Diagnostic Survey and examples of statements [137] characterizing each property. *(R)* stands for *Reverse* and indicates that the statement provides the opposite description of the property

Category	Property group	Property	Example of statement about the property
Essentials	Real Team	Bounded	*Team membership is quite clear—every member can identify exactly who is and isn’t on the team.*
		Stable	*The team is quite stable, with few changes in membership.*
		Interdependent	*Members of the team have to depend heavily on one another to get the team’s work done.*
	Compelling Direction	Clear	*There is great uncertainty and ambiguity about what the team is supposed to accomplish. (R)*
		Challenging	*The team’s purposes are so challenging that members have to stretch to accomplish them.*
		Consequential	*The team’s purposes are of great consequence for those served by the team.*
	Right People	Diversity	*Members of the team are too dissimilar to work together well. (R)*
		Skills	*Members of the team have more than enough talent and experience for the kind of work that the team does.*
Enablers	Sound Structure	Whole Task	*The team does a whole, identifiable piece of work.*
		Autonomy/Judgment	*The team work requires the members to make many “judgment calls” and take initiative as they carry it out.*
		Knowledge of Results	*Carrying out the team’s task automatically generates trustworthy indicators of how well the members are doing.*
		Team Size	*The team is just the right size to accomplish its purposes.*
		Team Norms	*It is clear what is—and what is not—acceptable member behavior in the team.*
	Supportive Context	Rewards/Recognition	*Excellent team performance is rewarded.*
		Information	*It is easy for the team to get any data or forecasts that members need to do their work.*
		Education/Consultation	*The team members do not receive adequate training for the work they have to do. (R)*
		Material Resources	*The team members can readily obtain all the material resources that they need for their work.*
	Coaching	Availability	*The team has readily available expert “coaches” who can help it learn from its successes and mistakes.*
		Helpfulness	*Coaches know how and when to intervene.*
Key Task Processes		Effort	*Members demonstrate their commitment to the team by putting in extra time and effort to help it succeed.*
		Strategy	*The team can come up with innovative ways of proceeding with the work.*
		Knowledge and Skill	*Members of the team actively share their special knowledge and expertise with one another.*

TDS measures six conditions of effective teams’ design [137], divided into *The Essentials* and *The Enablers*. The Essentials are the main conditions that result in a sturdy platform for an effective team. The Enablers are conditions that accelerate how fast teams grow into excellent performers. TDS also measures three *Key Task Processes* that emerge from the six conditions. These are meant to capture how well the team members are working together considering the extents of their capabilities.

TDS defines seven *measures of effectiveness* of teams. Three relate to *task performance*, including the satisfaction of the team’s users with the quality, quantity, and timeliness of the team’s work (*E1*, *Users satisfaction*), the appropriateness of the set of choices members make about how to carry out the work (*E2*, *Strategy appropriateness*), and whether the team brings ample and appropriate talent to bear on the work (*E3*, *Knowledge and skills*). The fourth measure concerns the quality of group processes and team interactions (*E4*, *Quality of group processes*). The remaining measures concern *member satisfaction*, including satisfaction with the relations between members (*E5*, *Satisfaction with relations*), team members’ opportunities to grow and learn over time (*E6*, *Growth opportunities*), and the general team member’ satisfaction (*E7*, *General satisfaction*).

### Study design

**Fig. 1:**
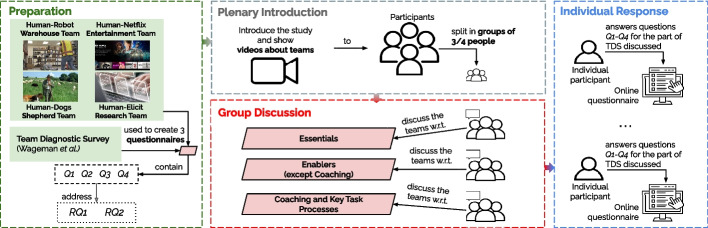
An overview of the key phases of our empirical study

Figure 1 illustrates the key phases of our study. In the preparation phase, we select four HI teams to provide contexts for discussions, and adapt TDS statements (see the last column in Table 1). In the plenary introduction, we introduce the selected HI teams and TDS statements to the participants. The participants discuss TDS statements with respect to the HI teams in the group discussion phase. Finally, the participants answer a TDS questionnaire, individually, via an online survey.

#### HI teams

TDS is used to evaluate a real team, which provides the necessary context to evaluate TDS statements. To create such contexts for our study, we formulate four HI teams.

*The Warehouse*: team consists of humans and robots collaborating to manage a warehouse by, e.g., restocking shelves, processing orders, and counting inventory.
*The Entertainment*: team consists of a person and an AI recommender (such as Netflix), interacting to find shows of interest to the person. Interactions take the form of the recommender advising the human, who responds with implicit feedback through selection or rejection of recommendations.
*The Research*: team consists of a researcher and an AI assistant (such as Elicit), interacting, via natural language and a GUI, to conduct research activities such as a literature review or writing a research proposal [11, 107].
*The Shepherd*: team consists of a shepherd and one or more shepherd dogs. Together they take care—guarding, moving, managing and controlling—of sheep.

Teams similar to the ones above exist in practice. This helps ground our study in realistic contexts. Whereas the first three teams are human-AI teams, the last team is a human-dog team, inspired by the ideas of using inter-species collaboration and interdependence as a metaphor for human-machine interaction [63, 87]. Besides being realistic, there is a large variety among these teams in terms of, e.g., application domain, team size and structure, types of AI (e.g., embodied vs. software agents), and types of interactions (simple clicks vs. natural language vs. multimodal). Having a variety of teams helps in eliciting a variety of perspectives.

#### TDS questionnaires

We formulate three questionnaires based on TDS—one for each category of properties in Table 1. We divide TDS into three parts to reduce the workload for the participants (each completing a different part of TDS) with an estimated completion time of 45 minutes. To allocate a similar workload to all participants, we move the *Coaching* property group from the *Enablers* to the *Key Task Processes* questionnaire. Each property is illustrated via a number of statements extracted from [137]. The full list of 61 statements used in our questionnaires can be found in our online appendix [29] (Table 1 reports examples).

The questionnaires contain three questions for each property. (*Q1*) Indicate how well each team reflects the statements concerning the property (5-point Likert scale). (*Q2*) Explain your evaluation (free text), clarifying what aspects were considered, whether a team had or lacked an important characteristic or process, whether the statements were difficult to evaluate, and whether the evaluation would have changed if a team exhibited a certain property or worked differently. (*Q3*) Indicate whether the statements are important for HI systems (yes/no). Finally, each questionnaire contains a question (*Q4*) Indicate the importance (5-point Likert scale) of the human teams’ effectiveness measures (*E1*–*E7* in Section 3.1) for HI systems.

#### Plenary introduction

The aim of this phase is to provide a good description of the HI teams and the questions that the participants need to answer. After an introduction about the study, the participants are shown short videos about the four HI teams, and the participants have an opportunity to ask for clarifications. The participants are asked to form groups of three or four people. Each group randomly receives one of the three types of questionnaires, ensuring a balanced distribution, and a printed version of the questionnaires and team descriptions (see online appendix [29]).

#### Group discussion

The aim of this phase is to stimulate the participants’ thoughts on both the HI teams and TDS questions assigned to them. The participants are asked to go through each property and each statement (see Table 1), and are encouraged to discuss concepts, example behaviors, and missing points. However, the participants are neither asked to answer the questionnaire as a group nor required to reach unanimous decisions.

#### Individual response

After the group discussion, participants are asked to individually fill out an online questionnaire on the TDS questions they discussed as a group. We opted for individual participant responses to enable the expression of individual opinions and to provide sufficient time to elaborate the responses.

### Study execution

The study was approved by Utrecht University Ethics assessment Committee. The participants were all researchers actively engaged in projects related to Hybrid Intelligence. All participants had at least an MSc degree. Their ages ranged from 23 to 65. All participants that took part in the Individual Response phase completed a consent form. Participants were not paid for their participation.

The Plenary introduction (approx. 30 minutes) and Group discussion (approx. 2.5 hours) phases of our study took place in May 2023. A total of 50 participants joined the Plenary introduction phase, which resulted in 15 groups (3-4 people each) in the Group discussion phase. The Individual response phase took place asynchronously during May and June 2023. 15 participants completed the Individual Response phase. Questions *Q1*-*Q3* were answered by five participants for each questionnaire. Therefore, for each questionnaire, we received feedback from participants of at least two different groups. Question *Q4* was answered by 15 participants.

### Study results

We analyze the study participants’ individual responses to answer the research questions posed earlier. To answer RQ1, we analyze the responses to questions *Q1*-*Q3*. To answer RQ2, we analyze the participants responses to *Q4*. We refer to participant quotes as P*i*, where *i* stands for the number of the participant who filled in the survey and whose quote we report. For each RQ, we mark main observations with a  to indicate properties of human teams that could be integrated into a quality model for HI teams. In contrast, observations marked with a  point toward potential challenges in extending the properties of human teams to HI teams.

#### RQ1 (Adequacy)

To answer RQ1, we focus on the properties of human teams that are considered in TDS. For each property, Table 2 shows, first, if the participants found the property important (*Q3*), second if the property was *well-understood* for HI teams (i.e., no difficulty was reported in *Q2* on the understandability of the property and its application to the HI teams for *Q1*), and finally, the mean and standard deviation of the scores each team obtained (*Q1*).

**Table 2:**
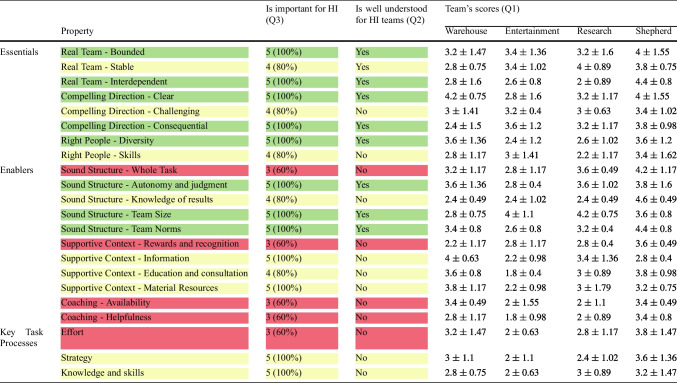
The number of participants considering a property important for HI (*Q3*), whether (yes/no) the participants reported difficulty in understanding a property (*Q2*), and the scores (mean ± SD) assigned by the participants to the four HI teams (*Q1*)

Each of the *Essentials* (the main conditions for a solid platform for human teams) was considered important for HI by *at least* 80% of the participants. Among the *Enablers* (team growth accelerators), the properties concerning a *Sound Structure* were considered important, but *Whole Task* scored lower (60%). Similarly, *Coaching* properties and *Rewards and Recognition* were on the 60% mark. Overall, all 22 properties were considered important for HI by at least 60% of the participants. Twelve properties (about half of all the properties) were considered important for HI by all the participants (100%).

**Table:** 

*Observation 1*
Each property from TDS was considered to be important for HI teams by the majority of the participants.

Although all properties have some importance for HI teams, not all are directly applicable in the way expressed in TDS. We discern directly applicable properties by identifying those deemed both *important* (*Q3*) and *well understood* by *all* the participants. We treat these properties as *directly applicable* to HI teams, and highlight them in green in Table 2. Yellow cells indicate properties that were considered important by at least 80% of the participants but not directly applicable, and the red cells indicate properties that received a mixed feedback about their importance.

**Table:** 

*Observation 2*
Properties concerning team structure, team composition, and team goals are well understood for HI teams.

**Table:** 

*Observation 3*
Eight properties characterizing effective human teams are both important and well understood for HI teams: bounded and interdependent team, right team size, diversity among members, a clear and consequential purpose, autonomy and judgment of members, and team norms.

**Fig. 2:**
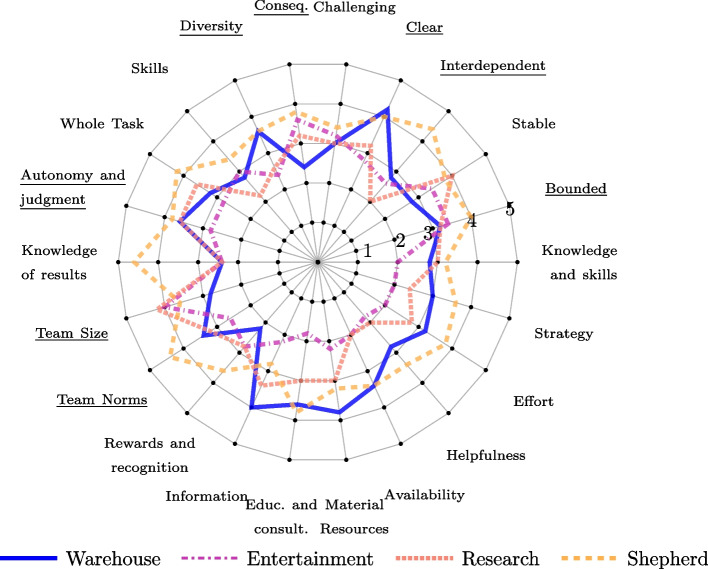
The mean scores on how well (*not at all* $=1$, *extremely well* $=5$) the HI teams reflect TDS properties (*Q1*)

To understand whether the eight properties in Observation 3 are well-exhibited in the considered HI teams, we examine the scores the teams obtained for these properties. These scores are shown in Table 2 and graphically illustrated in Fig. 2. Notably, of the eight properties (underlined in Fig. 2), none of the HI teams reflected six properties very well, i.e., they did not receive an average score higher than 4. The two exceptions are *Clear (Compelling Direction)* for which only the Warehouse team reported an average score of 4.2, and *Team Size*, for which the Entertainment and Research teams received an average score $\ge 4$.

The lack of clarity of direction stemmed from the AI’s limited context awareness (e.g., the weather might affect the type of movie to watch (P14), for the Entertainment team) and opacity regarding AI information utilization and optimization metrics (for both human and AI members) (P7). For *Team Size*, participants reported that 1-to-1 teams (Entertainment and Research) “*can hardly be larger*” (P5). However, these teams scored low on *Diversity*. Participants noted that, despite its importance for HI teams, there are contexts where “*diversity may not be needed*” (P11) or is less suitable (e.g., for physical work, or in 1-to-1 teams where the AI is optimized for one user (P7)).

An interesting case is that of the *Interdependent* property. Although it is considered both important and well understood for HI teams, participants noted that in all human-AI teams, “*humans can do their job without consulting the AI, while the AI cannot do any work on its own*” (P7). Differently, the Shepherd team was considered to reflect the property more than very well, in line with known knowledge about human-dog interaction [87].

It is also important to understand properties that were not found to be directly applicable or well-understood for HI. Nine properties (yellow in Table 2) are not directly applicable to characterize HI teams, although they were perceived as important for HI by at least 80% of the participants. Additionally, five properties (red in Table 2) are neither directly applicable nor clearly important for HI. These properties received mixed feedback about their importance.

Notably, all the properties with mixed evaluation about importance were also associated with difficulties in their understanding or evaluating them in relation to HI teams. In the following, we discuss the difficulties that the participants’ highlighted about these properties for HI teams, which lead us to the following observation.

**Table:** 

*Observation 4*
Properties concerning social or (inter-)personal aspects (with the exception of team norms), and team processes, including self-development, and control over team strategies, are less understood for HI teams.

Participants noted that statements describing five properties (from all TDS categories) made use of terminology that is either ambiguous or difficult to apply to non-human team members. This difficulty was raised, generally, with respect to the quantification of human-like abilities and behaviors that are not associated to current AI solutions. Specifically, the participants found it difficult to understand the following expressions when applied to the AI team members: *to fall into mindless routines* (*Strategy* property), *to share special knowledge* (*Knowledge and Skills*), *rewards and recognition* for AI members (*Rewards and recognition*), *stretching to accomplish goals* (*Challenging*), since “*AI either can (within small error rate) or cannot do something*” (P7), and *putting effort into the team* (*Effort*), since “*non-humans lack intrinsic motivation toward team success*” (P3).

**Table:** 

*Observation 5*
The meaning of human-centric terminology poses difficulties when referring to non-human team members.

Participants also reported various multifaceted difficulties broadly related to the asymmetry between humans and AI.

First, participants raised the question as to whether teams possess a certain property if it is only exhibited by human members. This issue became apparent in properties such as *Strategy* and *Knowledge and Skills*, for instance, concerning lessons learned from experience.

Second, the context dependency of certain properties emerged as a recurring challenge. For instance, participants observed that in some scenarios, e.g., in the Warehouse team, AI replacements don’t significantly impact (*Bounded*), whereas in others, e.g., Entertainment team, shared accounts or simultaneous usage (e.g., group movie watching) could alter team membership. Similarly, they noted that when dealing with HI systems, “*trustworthy indicators are not necessarily only related to performance*” (P12) and task execution (*Knowledge of results*), and that the importance of properties like *Autonomy and Judgment*, *Stability*, and *Team Size* and *Diversity* depends on the specific HI team and its objectives.

**Table:** 

*Observation 6*
Properties characterizing human teams do not fully address the inherent human-AI asymmetry of HI teams.

Some participants found that the *Coaching*-related properties did not necessarily apply to HI teams. Others could not link coaching to anything existing in current systems, except FAQs, help-desks, or tutorials, which “*still leave up to the human to learn how to use the AI better*” (P10). Some participants noted that *Reward and recognition* are “*possibly problematic, as the system can impose its task allocation and teamplay and not reward individual autonomy and creativity*” (P6).

**Table:** 

*Observation 7*
The relevance of coaching, and reward and recognition remains uncertain in the context of HI teams.

#### RQ2 (Effectiveness)

**Fig. 3:**
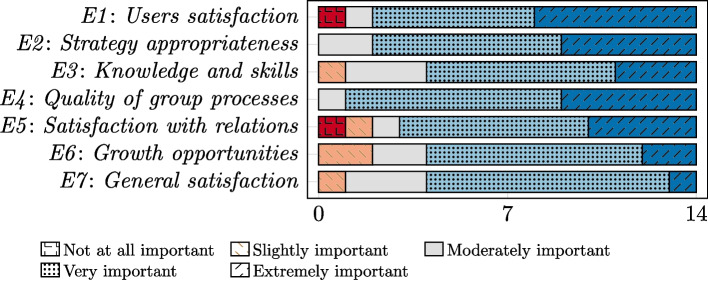
Evaluation of the importance of seven team effectiveness measures for HI teams (*Q4*)

An important aspect of TDS is measuring the effectiveness of human teams through seven measures (described in Section 3.1). Our study investigated their importance for HI teams (RQ2). Figure 3 shows stacked bar charts that illustrate the responses of 14 participants to question *Q4* which addresses RQ2 (one of the 15 total participants did not answer this question).

For each measure, the most common response was *Very important*. Each measure was considered at least *Moderately important* for measuring the effectiveness of HI teams by at least 85% of the 14 participants. Only one participant evaluated user satisfaction (*E1*) and satisfaction of members with their relations (*E5*) as *Not at all important*. The appropriateness of performance strategies (*E2*) and the quality of group processes and team interactions (*E4*) were considered at least moderately important by all the participants.

**Table:** 

*Observation 8*
Each measure of effectiveness of human teams from TDS is important for HI teams.

We observe that among all the measures, those considered less important by some participants (e.g., *E5* and *E6*) explicitly refer to social and human-oriented facets. For example, *E5* concerns members satisfaction with their relations with other members. *E6* is described as the opportunity for team members to grow and learn over time. This is in line with Observations 4-6, pointing to challenges in comprehending social aspects and human-centric language for non-human members, and in addressing the human-AI asymmetry.

## A quality model for HI teams

**Fig. 4:**
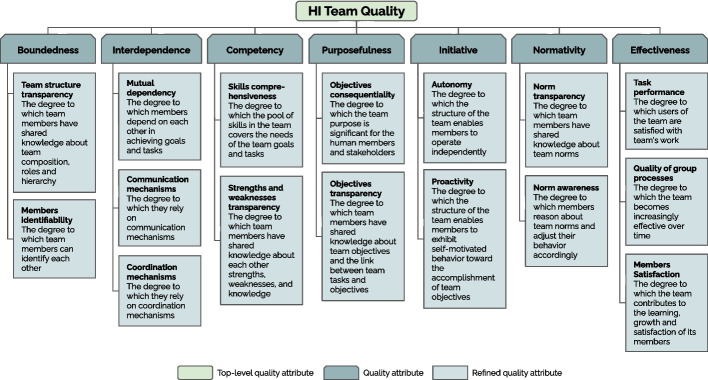
The recommended (though not exhaustive) quality attributes for HI teams

We leverage the observations and insights from the user study reported in Section 3.4 (RQ1 and RQ2) to lay the foundation for a quality model for HI teams. We focus on *quality attributes*, the primary elements of quality models [68, 126, 139]. A quality attribute represents a property, feature, or characteristic that affects a system’s quality. We propose seven quality *attributes* that summarize our findings and are based on the participants’ feedback, TDS statements (Table 1), and existing literature that supports our observations. More specifically, Observations 3 and 8 indicate that several properties of effective human teams were both considered important and well-understood by all the participants for HI teams and can be considered directly applicable to HI teams. Based on this, we propose the following set of high-level quality attributes of HI teams: *Boundedness*, *Interdependence*, *Competency*, *Purposefulness*, *Initiative*, *Normativity*, and *Effectiveness*. In the following, we elaborate further on them and the 16 lower-level quality attributes. These are also illustrated in Fig. 4 in a similar manner to [68]. In formulating the quality attributes, we acknowledge the inherent human-AI asymmetry, in line with Observation 6.

### Boundedness and interdependence

Wageman et al. [137] indicate that (human) teams work better when they are “real teams,” with clear boundaries, interdependent members, and stable membership, which gives members time and opportunity to learn how to work together well. The *Boundedness* and *Interdependence* attributes capture this also for HI teams. However, we do not propose stable membership as a standalone attribute for HI teams because in some cases AI members are replaceable (e.g., robots in a warehouse); instead, we integrate some of its aspects into *Boundedness*. We refine *Boundedness* into:

**Table:** 

*Team structure transparency*	The degree to which team members have shared knowledge about team composition, roles and hierarchy.
*Members identifiability*	The degree to which team members can identify each other.

These attributes underscore the need for clear roles in HI teams, an observation supported by our study participants and echoing the extensive literature on organization-aware multi-agent systems [3, 33, 71, 117, 135] where agents reason about their roles and responsibilities within a socio-technical organizational context. They also highlight that team members must identify each other to adjust expectations and personalize interactions. In this direction, participants questioned if algorithm updates constitute a team membership change “given that the [team] interactions may change dramatically”, similar to the ideas of compatibility of an AI update with prior user experience [4, 5].

We refine *Interdependence* into:

**Table:** 

*Mutual dependency*	The degree to which members depend on each other in achieving goals and tasks.
*Communication mechanisms*	The degree to which members rely on communication mechanisms in achieving goals and tasks.
*Coordination mechanisms*	The degree to which members rely on coordination mechanisms in achieving goals and tasks.

These attributes directly stem from TDS and align with extensive literature on interdependence and cooperation in both human and human-AI teams [13, 72, 98, 119]. For instance, Bradshaw et al. [11] identify interdependence, communication, and coordination devices as core elements for *joint activity* [78] in the context of adjustable autonomy and mixed-initiative interaction. Similarly, Johnson et al. [73] introduce coactive-design as a framework for designing human-robot systems that support interdependence by addressing observability, predictability, and directability requirements.

#### Competency and purposefulness

Team effectiveness, as highlighted by Wageman et al. [137], hinges on having the “right people”—those with necessary skills and diverse perspectives—and a compelling purpose. Our study participants noted that diversity, skills, and team size are interdependent in Human-AI (HI) teams. Participants also suggested revising TDS’s Skills property to explicitly acknowledge “strengths and weaknesses of each human or AI, and whether the team solves deficiencies”. Given these insights, we group these related properties under a new attribute, *Competency*, refined into:

**Table:** 

*Skills comprehensiveness*	The degree to which the pool of skills in the team covers the needs of the team goals and tasks.
*Strengths and weaknesses transparency*	The degree to which team members have shared knowledge about each other strengths, weaknesses, and knowledge.

The former emphasizes one of the key aspects underlying the ideas of Hybrid Intelligence, i.e., that of augmenting human intelligence by leveraging our strengths and compensating for our weaknesses [2]. The latter aligns with the ideas of transactive memory [66], which indicates that “knowing what other team members know” and being able to access this knowledge when needed, help assembling the distributed group knowledge into one coherent “group mind” that is associated with team effectiveness [8, 24].

Our user study confirmed that a compelling direction is essential for Human-AI (HI) teams. This directly informs our *Purposefulness* attribute, which ties together the Clear and Consequential Compelling Direction properties from TDS. We refine it into:

**Table:** 

*Objectives consequentiality*	The degree to which the team purpose is significant for the human members and stakeholders.
*Objectives transparency*	The degree to which team members have shared knowledge about team objectives and the link between team tasks and objectives.

Objective consequentiality emphasizes keeping humans at the center of HI teams and recognizing their place within broader socio-technical and socio-economic environments [35, 116]. Objective transparency is a need that was explicitly highlighted by some participants who mentioned the “knowledge asymmetry between AI and humans” in knowing when the team is doing well. This concern resonates with research on awareness requirements and runtime goal models [23, 27, 130], which offer AI mechanisms for self-reflection on system requirements and objectives.

#### Initiative and normativity

A sound team structure, as outlined in TDS, is fundamentally built upon core elements such as autonomy and judgment, a clear whole task, knowledge of results, and established team norms. These foundational aspects are crucial for team effectiveness. From TDS’s Autonomy property, we refine *Initiative* into two distinct quality attributes:

**Table:** 

*Autonomy*	The degree to which the structure of the team enables members to operate independently.
*Proactivity*	The degree to which the structure of the team enables members to exhibit self-motivated behavior toward the accomplishment of team objectives.

In an effective HI team, both human and AI members can demonstrate initiative and self-driven behavior to achieve team goals and objectives, solve problems, and prevent future problems [37]. It’s important to note that our focus here is on the team’s structure, rather than solely on the inherent traits of individual members. This distinction is vital because, in Human-AI (HI) teams, we often seek to maintain control over AI components, rather than granting them complete independence. This structural emphasis aligns with extensive literature on *adjustable* autonomy [17, 39, 67, 91, 103, 103] and proactivity [6, 112, 142], which frequently discuss scales and frameworks for managing control and initiative within human-AI systems.

In line with HI literature [2], some participants highlighted the importance of norm-aware AI members and of “well documented [and] acceptable behavior for AI”. From this, *Normativity* (following from Team Norms), is refined into:

**Table:** 

*Norm transparency*	The degree to which team members have shared knowledge about team norms.
*Norm awareness*	The degree to which members reason about team norms and adjust their behavior accordingly.

Effective team functioning hinges on a shared understanding of team ethics and norms [114], which directly impacts both intra-team trust and overall performance [55, 58]. For any team, including Human-AI teams, a common ethical code typically develops through team interactions over time and members continuously update their shared understanding of these norms and exhibit behavior that is both consistent with and justifiable by them [43, 59]. This aligns with extensive research in normative multi-agent systems [92], covering aspects like norm learning, representation, reasoning, and adaptation [9, 26, 128, 136], as well as literature on responsible AI, ethical reasoning and value-aligned design [1, 34, 64, 129].

#### Effectiveness

Observation 8 from our study, highlights the importance for HI teams of all the effectiveness measures mentioned in TDS. For this reason, in line with TDS, we refine *Effectiveness* into:

**Table:** 

*Task performance*	The degree to which users of the team are satisfied with the team’s work.
*Quality of group processes*	The degree to which the team becomes increasingly effective over time.
*Members satisfaction*	The degree to which the team contributes to the learning, growth and satisfaction of its members.

Task performance requires the team’s output to meet its *users* expectations, extending beyond direct team members. Quality of group processes emphasizes the need for a team to continuously learn, adapt, and improve its collaborative mechanisms over time. Finally, Members satisfaction underscores the importance of human members in HI teams and their growth and satisfaction over time.

## Evaluating quality attributes for HI teams

In this section, we report on an evaluation of the quality model for HI teams proposed in Section 4. More specifically, we investigate the understanding (RQ3) and relevance (RQ4) of the proposed quality attributes for HI teams in revealing HI team workings —specifically the benefits (or challenges) arising from their (lack of) consideration during the design of different types of HI systems.

### Method

We ran three Hybrid Intelligence (HI) competitions to collect data about quality-driven design of HI Teams.^2^ In each competition, participants formed teams of two to three members to explore the impact of quality attributes on HI use cases. Each participating team was provided with a description of four HI use cases (summarized in Table 3), as well as the quality attributes of HI teams described in Section 4. The four HI use cases included a description of the members of the HI team and their roles, the overall objective of the HI team, and a description of a specific task that the HI team was focusing on. This structure aimed at grounding the participants’ design activity in a clear, defined team context, emphasizing the interactions and shared goals central to the teaming perspective. To ensure the generality and robustness of our evaluation, we utilized different use cases than those used in the study described in Section 3.

**Table 3:** Overview of the use cases provided in the competitions

Use case	Description
*Research HI Team*	A pharmaceutical company forms a HI team consisting of a lead scientist and an AI-driven virtual agent. Their objective is to collaboratively analyze extensive scientific data to support drug and medication discovery. Their current task involves evaluating the inhibitory effect of a compound on neurodegeneration by reviewing literature, consulting experts, and drawing conclusions based on the evidence.
*Child Education HI Team*	In an advanced educational setting, a HI team comprising a remedial teacher, an educational therapist, an assistive robot, and a child with learning difficulties works together to develop a personalized learning program. The goal is to support the child’s learning by integrating professional expertise and robot-generated insights. Their current focus is on creating and adapting a learning plan for Alex, who faces auditory processing issues and dyslexia.
*Healthcare Diagnosis HI Team*	A HI team pioneers a collaborative approach to healthcare diagnostics. The goal is to accurately diagnose complex medical conditions and expedite assessments by intertwining human expertise and intuition with AI’s data-driven insights. Currently, the team is investigating Susan’s case, a middle-aged woman who suffers from a range of symptoms that none of the many doctors she has visited so far could make sense of.
*Cybersecurity Response HI Team*	This HI team, made up of cyber-security analysts and AI systems, focuses on detecting, analyzing, and counteracting cyber-threats. The team’s objective is to protect digital infrastructure by combining human expertise with AI’s capabilities in data analysis and threat detection. Their current mission involves mitigating denial-of-service attacks targeting government websites, traced to international sources beyond local jurisdiction.

Each participating team was asked to describe and submit two Usage Scenarios—narrative descriptions of how people or organizations interact with a system over time [57]. Usage Scenarios enable to gain insights into user needs and activities by describing key situations, events, or actions that occur during interactions. For our purposes, they represent a means to highlighting real-world activities, specific problems, conflicts, and trade-offs that map to our quality attributes. Furthermore, they support various phases of standard software and system development practices like requirements modeling and testing [49, 57]. Collecting a rich dataset of Usage Scenarios, therefore, is aligned with our ultimate objective of supporting the systematic and quality-driven design and evaluation of HI teams.

Each participating team submitted a Best Case Scenario and a Worst Case Scenario, illustrating the role of quality attributes on team collaboration in their selected HI team (use case). For each Usage Scenario, each participating team could choose one HI Use Case. In the best case, the participating teams described how effective collaboration and teamwork were achieved through the presence of at least one key quality attribute, explaining how this attribute—or a combination of attributes—contributed to a successful outcome. In the worst case, the participating teams described the challenges and risks that emerged when the HI team lacked one or more of these attributes, highlighting the negative impact on collaboration. Both scenarios were submitted in free-text format, with optional visual or multimedia elements to enhance clarity. To promote the quality of submissions, the top three teams were awarded a certificate and a pre-announced monetary prize. A jury, composed of the competition organizers, evaluated all submissions based on five criteria: creativity (original and innovative ideas), complexity (depth and sophistication of analysis), realism (practical scenarios and awareness of real-world constraints), potential impact on the use case (showcasing the impact of the quality attributes), and clarity of presentation. Participants received the detailed rubrics used to assess these criteria as part of the competition details, also available in our supplementary material [30]. We omit the competition results and winners here, as they are beyond the scope of this paper, but they can be found on the competition website: https://github.com/Hybrid-Intelligence-Competition.

By using the quality model to generate best and worst case scenarios, we observed participants’ understanding of the quality model (RQ3) and the relevance of each quality attribute in the generated scenario (RQ4). For each scenario, participants also explicitly evaluated the influence of the considered quality attributes on the outcome of the described scenario by providing a score using a five-point Likert scale: namely, *Not at all affected* (score 1), *Slightly affected* (score 2), *Moderately affected* (score 3), *Definitely affected* (score 4), or *Extremely affected* (score 5) by the attribute. Participants were required to indicate at least one attribute that either *Definitely affected* or *Extremely affected* each scenario. This constraint served to enforce the competition’s requirement to base the scenario on at least one quality attribute, thereby ensuring the scenarios were grounded in at least one attribute and allowing us to collect robust data relevant to the quality attributes. The participants were also asked to report additional attributes that they identified beyond those initially provided, if any.

Contrary to the study done in constructing the quality model (described in Section 3), the HI competitions targeted participants that had general AI knowledge, but not necessarily knowledge on hybrid intelligence. We repeated the competition three times; once in the conference in Autonomous Agents and Multiagent Systems (AAMAS) 2024, once in the conference in Hybrid Human-AI Intelligence (HHAI) 2024, and once in the authors’ Hybrid Intelligence Consortium (HIC).

The three competitions were approved by Utrecht University Ethics assessment Committee. All participants provided explicit consent for their data to be used in research publications and for their names, emails, and affiliations to be made public on the competition website to ensure proper credit for their scenarios. The detailed Participant Information Sheet and Consent Form can be found in our supplementary material [30].

**Table 4:** Demographics’ overview

Number of teams	24
Average team size	2.37 ± 0.56
Number of institutions	33
Number of participants	57
Participants’ age range	18-64
Ratio Male/Female/Non-Binary/Unknown	35/17/1/4

**Table 5:** The number of collected scenarios for each of the HI Use Cases

	Best Case Scenarios				Worst Case Scenario			
	HIC	AAMAS	HHAI	total	HIC	AAMAS	HHAI	total
T1. Research	1	3	0	4	1	1	0	2
T2. Education	4	3	5	12	2	2	3	7
T3. Healthcare	3	0	2	5	5	2	3	10
T4. Cybersecurity	2	1	0	3	2	2	1	5

#### Participants and collected data

##### Demographics

Table 4 presents a summary of the the demographics of the 24 participating teams. A total of 57 individuals participated in the competitions. We did not exclude participants who had previously been involved in the study described in Section 3, as the quality model used in the competitions was derived by the authors after the study, and the prior exposure to foundational concepts on human and hybrid teams was considered a form of purposive sampling. The distribution of participants by their reported level of education was as follows: less than a high school diploma (n=1), Bachelor’s degree (n=6), Master’s degree (n=21), Doctorate or professional degree (n=26), and prefer not to say (n=3). Regarding their self-reported familiarity with Hybrid Intelligence, the distribution was: not familiar at all (n=4), slightly familiar (n=8), moderately familiar (n=12), very familiar (n=15), extremely familiar (n=15), and prefer not to say (n=3). The participants’ self-reported areas of expertise spanned a range of computer science-related fields, as well as domains outside of computer science. Within computer science, these areas included: Artificial Intelligence (AI), Machine Learning (ML), Reinforcement Learning (RL), Human-AI/Robot/Computer Interaction and Communication, Robotics, Affective Computing, Emotion Recognition, Game Theory, Negotiation, Causality, Knowledge Representation, and Argumentation. Participants also reported expertise in fields beyond computer science, such as Robotic surgery and Agriculture.

##### Collected data

In total, we collected 48 free-text scenarios, 24 describing Best Case Scenarios and 24 describing Worst Case Scenarios. All the scenarios are available in our supplementary material [30]. Table 5 outlines the distribution of the scenarios across the different use cases and for the three competitions. In addition, our data collection for each scenario included the Likert scale ratings of the influence of the 16 proposed quality attributes described in Section 5.1, resulting in 48 individual scores for each attribute.

The use cases that were chosen the most often were the Child Education Team Use Case for describing a best case scenario, and the Healthcare Diagnosis Team Use Case for the worst case scenario. All use cases were selected by at least two teams across the different competitions.

#### Data analysis

**Fig. 5:**
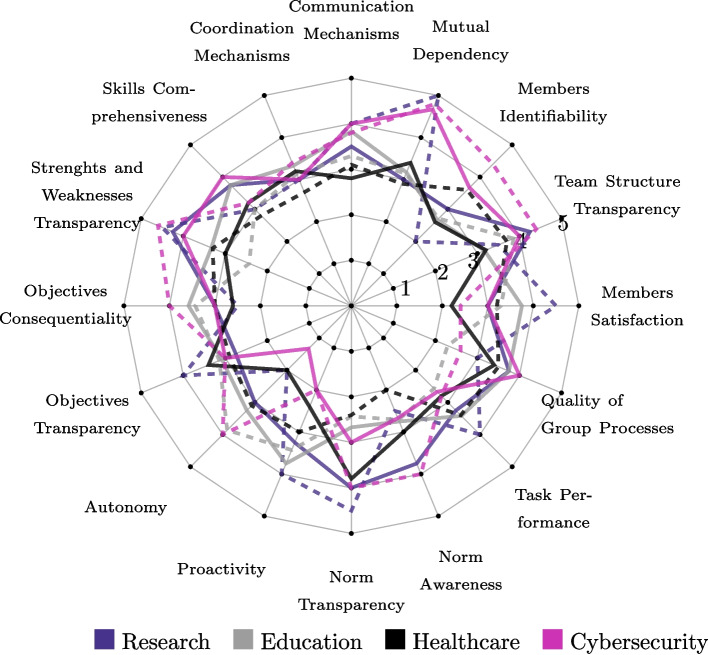
The mean scores on how much the outcome of the best case (solid lines) or worst case (dashed lines) scenarios is affected (*not at all* = 1, *extremely* = 5) by each of the quality attributes in the four different use cases

To address RQ3 (Understanding of the Quality Model) and RQ4 (Relevance of the Quality Attributes), we analyzed the scenarios collected through the competitions quantitatively and qualitatively. For the qualitative analysis, we conducted a codebook thematic analysis [15], which is positioned between reflexive and coding reliability approaches to thematic analysis. We adopted a combination of inductive and deductive orientation to data. The thematic analysis took place in an iterative fashion. First, two of the authors inductively identified evaluative statements in the scenarios generated by participants. These evaluative statements were then mapped to the quality model of Fig. 4. In cases in which the relation between the scenario and the quality model was provided by participants, we followed such mapping. When participants did not indicate the relation themselves, we conducted the mapping based on the strongest association between the scenario excerpt and the definition of each quality attribute. We report the qualitative results by illustrating the observations with quotes for the cases generate by participants. We refer to those cases as C*j*, where *j* refers to the number of the case. We complemented these insights with the quantitative analysis of the influence ratings provided by participants. Specifically, we inspected the mean and standard deviation that the participants attributed to the different quality attributes when indicating how much they thought the attributes affected the outcome of the scenario. Tables 6 and 7 report such mean (± standard deviation) scores. The average scores are also illustrated in Fig. 5 via a spider chart.

**Table 6:** Mean $\mu$ and standard deviation $\sigma$ scores (for each team) attributed by participants to each quality attributes when indicating how much they affected the outcome of the scenario (on a scale from 1 to 5, with 1 indicating *Not at all affected*, and 5 indicating *Extremely affected*)

	T1. Research				T2. Child Education				T3. Healthcare Diagnosis				T4. Cybersecurity Response			
	Best Case		Worst Case		Best Case		Worst Case		Best Case		Worst Case		Best Case		Worst Case	
Attribute	$\mu$	$\sigma$	$\mu$	$\sigma$	$\mu$	$\sigma$	$\mu$	$\sigma$	$\mu$	$\sigma$	$\mu$	$\sigma$	$\mu$	$\sigma$	$\mu$	$\sigma$
TST	4.25	0.96	3.5	0.71	3.17	1.4	3.86	1.46	3.2	2.05	3.7	1.57	4	1	4.4	0.89
MI	3	1.41	2	0	2.67	1.3	2.71	1.25	2.6	1.82	3.6	1.65	3.67	1.53	4.4	1.34
MD	3	1.83	5	0	3.17	1.7	3.14	1.77	3.4	1.52	2.9	1.6	4.67	0.58	4.8	0.45
CoM	3.5	1.73	4	1.41	3.83	1.47	3.29	1.7	2.8	1.64	3.1	1.45	4	1	3.8	1.64
CrM	3	1.41	3	1.41	3.33	1.37	3.14	1.57	3.2	1.3	2.7	1.34	3	1	3.4	1.14
SC	3.75	1.5	3	0	3.75	1.54	3	2	3.2	1.48	2.8	1.75	4	1	3.2	1.48
SW	4.25	0.5	4.5	0.71	3.33	1.23	2.43	1.4	3	1.87	3.3	1.7	4	1	4.6	0.55
OC	3	1.41	2.5	0.71	3.58	1.38	3.43	1.81	2.6	1.52	3	1.76	3	2	4	1
OT	2.75	0.96	4	1.41	3.08	1.62	3.14	1.35	3.4	1.52	2.9	1.45	3	1.73	3	1
A	3	1.41	2	0	3.25	1.29	3.86	0.69	2	1.41	3.1	1.6	1.33	0.58	4	1.73
P	3.25	1.26	4	1.41	3.75	1.14	3.43	1.72	2.4	1.14	3	1.56	2	1	2	1
NT	4	0.82	4.5	0.71	2.67	1.67	2.43	1.4	3.8	1.79	2.4	1.58	3	1.73	4	1
NA	3.75	0.96	2.5	0.71	2.75	1.76	2.71	1.7	3	2	2	1.33	2.67	2.08	4	1
TP	3.25	1.71	4	1.41	3.42	1.68	2.57	1.51	2.8	1.1	3.4	1.78	2.67	2.08	2.8	2.05
QP	3.75	0.96	3	1.41	3.75	1.54	2.29	1.7	3.4	1.67	3.5	1.84	4	1.73	2.6	1.82
MS	3	0.82	4.5	0.71	3.75	1.54	3.29	1.7	2.2	1.1	3.2	1.81	3	1.73	2.4	1.34
average	3.41	1.23	3.5	0.8	3.33	1.48	3.05	1.55	2.94	1.56	3.04	1.61	3.25	1.36	3.59	1.21

**Table 7:** Mean $\mu$ and standard deviation $\sigma$ scores (across all teams) attributed by participants to each quality attributes when indicating how much they affected the outcome of the scenario (on a scale from 1 to 5, with 1 indicating *Not at all affected*, and 5 indicating *Extremely affected*)

	Average All Teams			
	Best Case		Worst Case	
Attribute	$\mu$	$\sigma$	$\mu$	$\sigma$
Team Structure Transparency (TST)	3.66	1.35	3.87	1.16
Members Identifiability (MI)	2.99	1.52	3.18	1.06
Mutual Dependency (MD)	3.56	1.41	3.96	0.96
Communication Mechanisms (CoM)	3.53	1.46	3.55	1.55
Coordination Mechanisms (CrM)	3.13	1.27	3.06	1.37
Skills Comprehensiveness (SC)	3.68	1.38	3.00	1.31
Strengths and Weaknesses transparency (SW)	3.65	1.15	3.71	1.09
Objectives consequentiality (OC)	3.05	1.58	3.23	1.32
Objectives transparency (OT)	3.06	1.46	3.26	1.30
Autonomy (A)	2.40	1.17	3.24	1.01
Proactivity (P)	2.85	1.14	3.11	1.42
Norm Transparency (NT)	3.37	1.50	3.33	1.17
Norm Awareness (NA)	3.04	1.70	2.80	1.19
Task Performance (TP)	3.04	1.64	3.19	1.69
Quality of group processes (QP)	3.73	1.48	2.85	1.69
Members Satisfaction (MS)	2.99	1.30	3.35	1.39
average	3.23	1.41	3.29	1.29

### RQ3 (Understanding) and RQ4 (Relevance)

All quality attributes were considered to affect the outcome of either the best- or the worst-case scenario across all use cases by at least one team. This can be seen by the lack of quality attributes with a mean value of 1 (default value in the Survey, indicating *Not at all affected*) across use cases in Tables 6 and 7. In the best-case scenario, four quality attributes had a mean above 3.6, between *Moderately affected* and *Definitely affected* (Table 7). These were *Team Structure Transparency* (*M* = 3.66, *SD* = 1.35), *Skills Comprehensiveness* (*M* = 3.68, *SD* = 1.38), *Strengths and Weaknesses* (*M* = 3.65, *SD* = 1.15), and *Quality of group processes* (*M* = 3.73, *SD* = 1.48). These were considered to affect the best-case scenarios at least slightly (score $\ge 2$) in 19 out of 24 cases ($79.2\%$) for *Team Structure Transparency*, and 21/24 ($87.5\%$) for the other three attributes. In the worst-case scenario, three quality attributes had a mean above 3.6, between *Moderately affected* and *Definitely affected* (Table 7). These were *Team Structure Transparency* (*M* = 3.87, *SD* = 1.16), *Mutual Dependency* (*M* = 3.96, *SD* = 0.96), and *Strengths and Weaknesses* (*M* = 3.71, *SD* = 1.09). These were considered to affect the worst-case scenarios at least slightly (score $\ge 2$) in 21 out of 24 cases ($87.5\%$) for *Team Structure Transparency*, 19/24 ($79.2\%$) for *Mutual Dependency*, and 18/24 ($75\%$) for *Strengths and Weaknesses*.

#### Boundedness and interdependence

The scenarios generated by participants related the attribute *Team Structure Transparency* with clarity in role definition and team member capabilities. For best-case scenarios, this leads to ensuring that *“each member can focus on tasks that best suit their expertise”* (C21), whereas in worst-case scenarios, a lack of clear role definitions might lead to *“confusion over task ownership and responsibilities”* (C22). *Team Structure Transparency* was considered especially relevant in dyadic interactions, scoring high in the best case scenario in the research scenario (*M* = 4.25, *SD* = 0.96), and in the cybersecurity scenario both in the best- (*M* = 4, *SD* = 1) and worst-case scenarios (*M* = 4.4, *SD* = 0.89) –see Table 6. In these cases (i.e., research, cybersecurity), *Team Structure Transparency* relied on clear task decomposition for correctly assigning the task to a team member.

**Table:** 

*Finding 1*
Transparency over team structure, membership, skills and weaknesses, and objectives were seen as means to optimal task delegation.

Similarly to *Team Structure Transparency*, the attribute *Member Identifiability* was often linked with clarity about team membership for task decomposition; i.e., knowing the expertise of each team member. For the best-case scenario, this requires *“to have a good idea both of the AI system and its limitations and capabilities”* (C25). In the worst-case scenario, participants highlighted that the lack of identification of the contribution and responsibilities of each team member can lead to *“critical tasks being neglected or improperly handled”* (C22).

When dealing with the attribute *Mutual Dependency*, participants referred to the mutual augmentation of capabilities between humans and AI, where *“AI alone or human alone would not be able to do it by themselves in this context”* (C17). The effect of *Mutual Dependency* on the best-case scenario was translated into appropriate reliance on the unique capabilities of each team member. In the worst-case scenario, participants referred to *“an imbalance in dependency”* (C2), where mutual dependency was mishandled leading humans to rely on AI without appropriate checks. For the worst-case scenario in the use case of cybersecurity, *Mutual Dependency* was also seen as a burden that might slow down decision-making in a time-sensitive context.

The attribute *Communication Mechanisms* was deemed crucial to avoid misunderstandings. For an effective collaboration, *“both agents need to be extremely clear on their communication and explanation for their decisions”* (C13). The mismanagement of communication protocols was considered to be key in the worst-case scenario in the research use case, where unclear protocols would lead to delays in information sharing.

The attribute *Coordination Mechanisms* was understood as the means for the team to synchronize their actions and progress in their tasks for the best-case scenario. In the worst-case scenario poor coordination was understood as the isolated performance of different team members, *“a fragmented approach”* (C40) that might lead to confusion.

**Table:** 

*Finding 2*
Communication and coordination mechanisms were seen as key attributes to properly manage dependencies between humans and AI actors.

#### Competency and purposefulness

The attribute *Skill Comprehensiveness* was mainly understood as knowledge complementarity. For the cybersecurity use case, *Skill Comprehensiveness* was considered to be especially important in the best-case scenario to help *“cover as many potential scenarios”* (C27). In the worst-case scenario of this same use case, *Skill Comprehensiveness* was understood as skill multiplicity rather than complementarity. Members were perceived to duplicate efforts unnecessarily.

Having transparency over the *Strengths and Weaknesses* of each team member was considered highly relevant in the research and cybersecurity use cases for both the best-case scenario (*M* = 4.25, *SD* = 0.5 for research, *M* = 4, *SD* = 1 for cybersecurity) and worst-case scenario (*M* = 4.5, *SD* = 0.71 for research, *M* = 4.6, *SD* = 0.55 for cybersecurity). In the best-case scenario, this allows optimal task delegation —similar to *Team Structure Transparency*. In the worst-case scenario, a lack of transparency in each team members’ capabilities might hinder effective collaboration since humans might be blindsided as to what the AI can or cannot do (C14). Transparency over skills was often seen as a means to other quality attributes, such as the correct management of mutual dependencies.

*Objectives consequentiality* was considered especially relevant in the worst-case scenario of the cybersecurity use case (*M* = 4, *SD* = 1) – see Table 6. The scenarios described for such case indicate that participants understood the implications of the objectives and possibly risky outcomes as the stakes of the task. The implications of the task were perceived to be dependent on how the team coordinate their efforts.

*Objectives Transparency* was understood as clarity and alignment in team’s objectives and efforts. Clear articulation of objectives enable *“both team members [to] understand the ultimate goal of their work”* (C21). For the research use case, having a shared goal and transparency over the objective that is being pursued might have been perceived as a natural element of teamwork: it was deemed especially relevant for the worst-case scenario (*M* = 4, *SD* = 1.41), yet it did not greatly affect the best-case scenario (*M* = 2.75, *SD* = 0.96).

**Table:** 

*Finding 3*
Communication mechanisms, objectives transparency and autonomy were perceived to be natural elements of teamwork. Their impact on the bestcase scenario was limited, yet their absence contributed considerably to the worst-case scenario.

#### Initiative and normativity

Participants mostly referred to AI autonomy, rather than the *Autonomy* that the team structure provided to human and AI team members. In best-case scenarios, *Autonomy* received the lowest average score (*M* = 2.40, *SD* = 1.17), indicating that the attribute was considered on average to affect the scenarios between *slightly* and *moderately* –see Table 7. In the best-case scenarios generated by participants the impact of *Autonomy* was generally not mentioned. However, in the worst-case scenario, participants referred to *“misplaced autonomy”* (C22) as a factor contributing to AI being unable to correct itself. The effect of *Autonomy* on the worst-case scenario differed per use case. For the research use case, humans were seen able to correct AI if adequate communication mechanisms were in place, and autonomy was, therefore, not considered as an impactful factor. Instead, for the cybersecurity use case, lack of AI autonomy was perceived to lead to non-optimal usage of resources. This might have been caused by the time-sensitive nature of the cybersecurity use case. Similarly to *Autonomy*, participants mostly referred to AI proactivity and not to the *Proactivity* of human members. The way the team was defined was perceived to condition the level of *Proactivity* of the AI. The effect of AI proactivity on the resulting team performance was, according to the generated scenarios, limited.

**Table:** 

*Finding 4*
Asymmetry between humans and AI was apparent when assessing autonomy and proactivity. Autonomy and proactivity were mostly evaluated with respect to the AI, but not with respect to human members.

When reflecting on *Norm Transparency*, participants referred to standards of practice as well as *“ethical [and] procedural norms”* (C30). Such standards could involve guidelines about *“data integrity and source validation”* (C2). For the healthcare use case, for example, participants highlighted the need to shed light onto the medical norms that govern the AI behavior. A temporal dimension was also mentioned, where AI systems like the educational robot should be aware of the change in norms when interacting with patients over time. *Norm Awareness* was perceived similarly to *Norm Transparency*. Both quality attributes were often reported jointly. *Norm Awareness* scored lowest in the worst-case scenario (*M* = 2.80, *SD* = 1.19), indicating that on average this attribute affected the outcome of the worst case scenarios only slightly (Table 7).

**Fig. 6:**
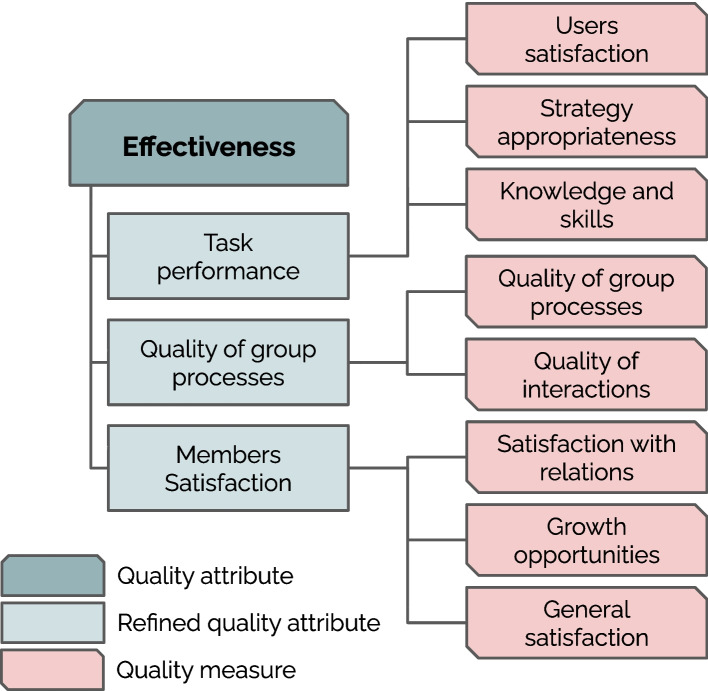
A summary of the recommended (though not exhaustive) quality measures for effectiveness attributes of HI teams

#### Effectiveness

Effectiveness attributes refer to the way interactions over time affect the current performance of the team, i.e., the extent to which *Task Performance*, *Quality of Processes* and *Member Satisfaction* are contributing factors towards the current interaction. For instance, *Task Performance* was considered to depend on data reliability. *Quality of Group Processes* was mainly interpreted as a consequence of team work, rather than a contributing factor. Similarly, participants referred to *Member Satisfaction* by acknowledging frustration and high dissatisfaction of team members in the worst-case scenario and the opposite in the best-case scenario. From participants’ scenarios, and as opposed to the rest of quality attributes, effectiveness attributes appear to be understood as related to the outcome of the HI teams, rather than the process.

**Table:** 

*Finding 5*
Effectiveness attributes were perceived as orthogonal to the other quality attributes. They were mostly evaluated as a consequence of the best- and worst-case scenarios, rather than factors contributing to those scenarios.

## Discussion and conclusions

### Summary of findings

In this paper, we proposed a team-oriented approach to HI, framing HI systems as HI teams. In Section 3, we investigated, through a user study involving 50 HI researchers, the adequacy and importance of human team properties and effectiveness measures (RQ1 and RQ2), as described in the Team Diagnostic Survey (TDS) [137], to characterize HI teams.

Results highlighted the importance of several of these properties and effectiveness measures for HI teams, and the need for further research into addressing AI-human knowledge asymmetry, promoting hybrid teams interdependence, and integrating social or (inter-)personal factors in AI decision-making. Further, our study highlighted some limitations of TDS when applied to hybrid teams. These included interpreting human-centric terminology for non-human team members, and the difficulty in addressing the inherent asymmetry between humans and AI.

Based on these findings, in Section 4, we proposed a quality model for HI teams, identifying seven high-level *quality attributes* — including, boundedness, interdependence, competency, purposefulness, initiative, normativity, and effectiveness. These quality attributes for HI teams put a particular emphasis on the system-level perspective to HI team qualities, on the human-AI asymmetry, and on the need for *shared knowledge* among team members. According to our model, and in line with literature on shared mental models and team processes [56, 74, 79, 93, 95, 121], shared knowledge is required to be preserved, leveraged, and kept up to date about several aspects, including the structure of the team, the strengths and weaknesses of the team members, the team’s objectives, and the team norms.

Since the concept of “quality” highly depends on the application context and concrete use case [126], we proposed identifying and tailoring distinct quality attributes to different types of HI team to acknowledge their unique characteristics and goals. This means a quality model for HI teams should address human-AI asymmetry and team specific traits, and the relevance of quality attributes should vary by teams. For example, *Members identifiability* might apply to robots but not to human team members in a warehouse, unless robots have unique characteristics. Likewise, recognition and coaching might be relevant for human team members but not for non-human counterparts (see Observation 7). TDS’s Strategy property could lead to a quality attribute related to *adaptivity*, potentially only applicable to human team members due to the current AI systems limited flexibility in innovating work approaches.

In Section 5, we evaluated the understanding and relevance of the proposed quality attributes (RQ3 and RQ4) by quantitatively and qualitatively analyzing 48 scenarios that we collected via three competitions at scientific events and that we openly release. The evaluation showed that all dimensions of the quality model were, to some extent, considered relevant. While this does not mean that the current form of the quality model is final, it does suggest that the attributes in the model are appropriate for capturing the quality of HI systems across use cases, and confirm our recommendations on acknowledging the human-AI asymmetry and team specific traits.

The evaluation of the quality model also clearly illustrated the importance of the proposed quality attributes by highlighting the understanding of benefits and challenges arising from (the lack of) their consideration during the design of HI systems. Our analysis leads us to the following conclusions and take-home messages for HI researchers to use the quality model to guide the design of HI systems:

1. Since all the quality attributes were considered relevant, HI researcher should account for all of them when designing HI systems and explore additional ones that might be needed for their specific use case.
2. Attributes dealing with transparency and shared knowledge (e.g., Team Structure Transparency, Member Identifiability, Skills and Weaknesses Transparency, and Objectives Transparency) or with communication and coordination mechanisms, should be treated as *affordances* of HI systems whose design should lead to the proper functioning of the team (e.g., optimal task delegation, proper management of dependencies).
3. Special attention should be placed on the design of communication mechanisms, objectives transparency, and mechanisms to adjust the level of autonomy of team members: these are perceived to be natural elements of teamwork and as key factors that can lead to worst-case scenarios.
4. Designers of HI systems shall be aware of the tendency of reducing autonomy and proactivity attributes to the AI system, and actively account for the level of autonomy and proactivity that the team structure provides also to human team members. This is key in determining human team members’ level of satisfaction and the team success in the long-term.
5. Effectiveness measures were perceived as the consequence of teamwork rather than factors characterizing it. As such, they should be treated as orthogonal to the rest of attributes when characterizing HI teams or when designing experimental setups.

### Limitations and future directions

By emphasizing a system-level, human-AI perspective, our quality model for HI teams provides an alternative perspective to existing technology-oriented taxonomies of HI and to existing AI quality models. Our paper, therefore, conceptually defines dimensions that researchers working on hybrid intelligence should account for when designing or evaluating their systems. The quality attributes described in our quality model were synthesized using the human team properties described in the TDS as a starting point. Further work is needed to consider alternative team models (e.g., [133]) and additional attributes that specific contexts that were not explored in this paper might require. These can highlight additional relevant dimensions of HI teams, including aspects such as team cohesion [65, 105, 134], group engagement [109], and measures beyond effectiveness such as team process measures [76], privacy [44, 84, 102], creativity [25, 104, 120] and dominance [70]. We emphasize that our quality model serves as a design and evaluation guide for Human-AI (HI) teams, and is not intended as a new theoretical framework for team behavior. It does not constitute an alternative to established descriptive and conceptual models of teams, such as the TDS or the Input-Mediator-Output (IMO) family of models, which aim to explain team functioning. The development of a comprehensive theoretical model of HI teams remains a vital, yet distinct, direction for future research.

An important limitation of our study concerns the limited number of participants. For answering research questions RQ1 and RQ2 (Section 3), we sought HI researchers as subjects since developing a quality model for an emerging concept like HI requires both the subject knowledge, and the willingness and time to engage in in-depth discussions. Finding such subjects is challenging. As evident from our experience, although we started with 50 researchers, eventually only 15 researchers completed the study (5 per section of the TDS). Yet, we synthesized interesting observations and recommendations, systematically, which led to the development of our quality model.

For answering research questions RQ4 and RQ3 (Section 5), we targeted participants with general AI knowledge. While this enabled us to evaluate the relevance and understanding of the quality attributes through the analysis of 48 different scenarios designed by a total of 57 participants, the sample can still be considered limited. The majority of the participants of our studies are related to the Hybrid Intelligence community (e.g., more than half of the respondents in Study 2 indicated to be at least very familiar with Hybrid Intelligence). The proposed quality model should also be validated with experts from adjacent research and industry communities related to AI, such as practitioners working in the areas of Human-Robot Interaction, Human-AI Interaction, or Agentic AI. While we believe that our work can be valuable and relevant to these communities, we invite them to use the quality model in their practices and we welcome improvements as new insights emerge.

Our findings should also be seen in light of the current state of AI and the speed in which AI develops. Some of our participants realised that changing the algorithms of non-human members might affect teamwork. We also observed that among all the measures, those considered less important by some participants (e.g., *E5* and *E6*) explicitly refer to social and human-oriented facets. And Observations 4-6 point to challenges in comprehending social aspects and human-centric language for non-human members, and in addressing the human-AI asymmetry. We advocate that our study should be done again as AI capabilities progress. In the future, it may no longer be the case that the non-human member simply can or cannot do a task, but it might become a matter of mutual learning and joint decision-making on how the task is performed and by which team member. At some point in time, even reward, recognition, replaceability and identifiability of the non-human members might become more relevant.

One of our findings suggests that effectiveness of the HI team is related to outcome rather than process. This finding might have been different if the participants would have been part of the HI system. We intend to conduct similar evaluations to those reported in this paper, with the fundamental difference that the participants would themselves be the human members of the HI team tested. We hypothesize that this might affect their view on the social and human-oriented facets of the HI Quality model, and their view on the importance of the process versus the outcome when thinking about the HI team’s effectiveness.

Finally, having a comprehensive quality model for HI teams is not only important as a tool for designing new HI systems with quality in mind, but also useful for assessing existing HI systems. For a quality model to become useful for performing quality evaluations of such teams, an essential step is establishing quality measures that can be used to evaluate the different quality attributes. While in this paper we mainly focused on quality attributes, our explicit investigation on effectiveness measures (RQ2, Section 3.4.2) suggests that quality measures for the Effectiveness quality attributes should include (see Section 3.1 for more details): *users satisfaction*, *strategy appropriateness*, *knowledge and skills*, *quality of group processes* and of *interactions*, *satisfaction with relations*, *growth opportunities*, *general satisfaction*. Figure 6 illustrates how these measures relate to the different effectiveness quality attributes of our quality model. We stress that none of these measures are exclusive to the AI component. This reinforces our recommendation that measures for HI teams should explicitly encompass the entire team and focus on the quality of interactions and dynamics among its members. In future work, we intend to investigate appropriate measures for the evaluation of the other proposed quality attributes, providing a comprehensive quality model for HI practitioners and settings in which participants are the human members of the HI teams in which the measures and attributes of the HI Quality model are tested. This will lead to the realization of a HI-Team diagnostic tool for quantitative assessment of HI teams (in line with [69]), and effective visualization strategies (e.g., Fig. 2) for comparing HI teams over multiple dimensions.

## Data Availability

The data generated and/or analyzed during the first empirical study (RQ1 and RQ2) is available in the Zenodo repository https://doi.org/10.5281/zenodo.8400166. The data generated and/or analyzed during the second empirical investigation (RQ3 and RQ4), including the data set of 48 scenarios collected during the Hybrid Intelligence competitions, is available in the Zenodo repository https://doi.org/10.5281/zenodo.15640622.

## Footnotes

1 https://github.com/Hybrid-Intelligence-Competition

2 Please refer to the supplementary material for the exact description and competitions details [30].

## Code Availability

The source code generated to analyze data during the current study is available in the Zenodo repository https://doi.org/10.5281/zenodo.15640622.
